# Contralateral knee hyperextension is associated with increased anterior tibial translation and fewer meniscal injuries in the anterior cruciate ligament-injured knee

**DOI:** 10.1007/s00167-018-5047-7

**Published:** 2018-07-04

**Authors:** David Sundemo, Christina Mikkelsen, Riccardo Cristiani, Magnus Forssblad, Eric Hamrin Senorski, Eleonor Svantesson, Kristian Samuelsson, Anders Stålman

**Affiliations:** 1 Department of Orthopaedics, Institute of Clinical Sciences, The Sahlgrenska Academy, University of Gothenburg, Gothenburg, Sweden; 2 Department of Orthopaedics, Sahlgrenska University Hospital, Mölndal, Sweden; 3 Capio Artro Clinic, Sophiahemmet, Stockholm, Sweden; 4 Department of Molecular Medicine and Surgery, Stockholm Sports Trauma Research Center, Karolinska Institutet, Stockholm, Sweden; 5 Department of Health and Rehabilitation, Institute of Neuroscience and Physiology, The Sahlgrenska Academy, University of Gothenburg, Gothenburg, Sweden

**Keywords:** Knee hyperextension, Anterior cruciate ligament reconstruction,, Generalized joint hypermobility, Generalized joint laxity, KT-1000, Anterior knee laxity, Anterior tibial translation

## Abstract

**Purpose:**

To investigate the influence of hyperextension of the contralateral healthy knee on anterior tibial translation (ATT) and the presence of associated injuries in the anterior cruciate ligament (ACL)-injured knee.

**Methods:**

A local patient data register containing the surgical and clinical data of patients undergoing ACL reconstruction was analyzed. Patients were divided into groups according to the degree of hyperextension of the contralateral knee: normal (Group A ≤ 0°), mild (Group B 1°–5°), moderate (Group C 6°–10°), and severe (Group D > 10°). The ATT was measured in both knees preoperatively and 6 months postoperatively using the KT-1000 arthrometer. The presence of associated meniscal and cartilage injuries was noted. Using multivariate analysis, Groups B, C, and D were compared with Group A, using this group as a reference.

**Results:**

A total of 10,957 patients were available in the register and 8502 (Group A *n* = 4335, Group B *n* = 3331, Group C *n* = 771, Group D *n* = 65) were included in the final analysis. Groups B (10.3 mm; 95% CI 0.06–0.042, *p* < 0.0001) and C (10.6 mm; 95% CI 0.23–0.89, *p* = 0.006) showed significantly greater preoperative ATT in the injured knee compared with the control group (10.1 mm). Moreover, at the 6-month follow-up, greater ATT was observed for Groups B (8.5 mm; 95% CI 0.13–0.45, *p* < 0.0001), C (8.5 mm; 95% CI 0.02–0.60, *p* = 0.035), and D (9.1 mm; 95% CI − 0.08–1.77, *p* = 0.082) compared with Group A (8.2 mm). Meniscal injuries were less frequent in patients with contralateral hyperextension [Group B 903 (27.1%) *p* < 0.0001, Group C 208 (27.0%) *p* = 0.0003, and Group D 12 (18.5%), 0.012] compared with the control group [Group A 1479 (34.1%)].

**Conclusion:**

Contralateral knee hyperextension is associated with greater pre- and postoperative ATT in the ACL-injured knee. In patients with contralateral knee hyperextension, concomitant injuries to the menisci are less frequent. Surgeons should consider grafts with superior properties regarding postoperative anteroposterior laxity to patients with contralateral knee hyperextension.

**Level of evidence:**

Retrospective cohort study, Level IV

## Introduction

To enhance our understanding of ACL injury causality, the risk factors for primary ACL injury and re-rupture have been the subject of vigorous interest in the research community during the last decade [1–3, 25]. Among other factors, generalized joint hypermobility (GJH) and knee hyperextension have been found to be significant risk factors for sustaining a primary ACL injury in recent studies [18, 19, 21, 27]. Patients with hypermobility also run an increased risk of graft rupture and contralateral ACL rupture, as well as reporting inferior subjective outcome [13, 15]. Moreover, it has been suggested that knee hyperextension alone, without considering GJH, results in poorer clinical and patient-reported outcomes after ACL reconstruction [14]. The exact mechanism behind this connection is not known.

Generalized joint hypermobility is diagnosed using the Beighton Hypermobility Score [22]. Generalized joint hypermobility is merely a definition of the hypermobility of the synovial joints and is asymptomatic. If accompanied by arthralgia or other symptoms, it is instead part of a syndrome [20]. There is an overlap between GJH and connective tissue disorders like Joint Hypermobility Syndrome, Ehlers–Danlos Syndrome—Hypermobility Type, and also with rare hereditary afflictions like Marfan’s syndrome, osteogeneisis imperfecta, or other subtypes of the Ehler–Danlos spectrum [6]. There is diversity in the genetic causes of the above-mentioned syndromes, although deficiency of the connective tissues is regarded as a mutual biological cause [20]. The Beighton Hypermobility Score assesses particular synovial joints [4]. Knee hyperextension is an important part of GJH and its existence gives an indication of possible changes in connective tissue aggradation.

It has been shown that patients with knee hyperextension undergoing ACL reconstruction run an elevated risk of graft impingement [9, 16], an outcome that can cause graft rupture and joint instability [7, 8]. ACL impingement has been shown to increase linearly with increased knee extension, even in non-injured knees [10]. In spite of this, in a recent publication, hyperextension of the ACL-injured knee was not found to be predictive of either increased anterior tibial translation (ATT) or subsequent graft tear [5]. In the ACL-injured knee, however, concomitant cartilage or meniscus injuries can interfere with the range of motion, thereby possibly underestimating the preinjury degree of knee hyperextension in the ACL-injured knee. Moreover, the relationship between knee hyperextension and concomitant injuries to the menisci or cartilage is unclear. However, recent studies have not observed a significant effect on the frequency of meniscal or chondral injuries in patients with joint hypermobility [15, 26]. To our knowledge, no analyses of the degree of hyperextension in the contralateral knee with regard to ATT or concomitant meniscal or cartilage injuries have previously been conducted.

The main purpose of this study was to determine the association between hyperextension of the contralateral healthy knee and increased ATT in the ACL-injured knee. The second purpose of the study was to investigate the potential relationship between contralateral knee hyperextension and concomitant cartilage and meniscal injuries. It was hypothesized that increased hyperextension of the contralateral knee would be associated with an increase in ATT and with an increase in the frequency of concomitant injuries to the ACL-injured knee.

## Materials and methods

Using a retrospective study design, a total of 10,957 patients who underwent ACL reconstruction between February 1990 and December 2015 at the Capio Artro Clinic, Stockholm, Sweden were assessed for inclusion. Patients aged 14 or older who underwent ACL reconstruction using either a patellar tendon (PT) or a hamstring tendon (HT) autograft were eligible for inclusion. Patients who had suffered a previous ipsilateral or contralateral ACL injury were excluded from the study. Meniscal or articular cartilage injuries did not disqualify patients from inclusion.

### Surgical technique

The PT ACL reconstructions were performed by harvesting the central third of the patellar tendon with two bone blocks. The graft was fixed at both the tibial and the femoral sides using interference screws (Softsilk, Smith and Nephew, Andover, Mass, USA) or using an Endobutton fixation device (Smith and Nephew, Andover, Mass, USA) on the femoral side. The HT reconstructions were performed using a triple or quadruple semitendinosus tendon autograft. A supplementary gracilis tendon could be used and incorporated if the width of the semitendinosus graft was considered insufficient. The graft was fixed with an Endobutton fixation device on the femoral side and Ultrabraid (Smith and Nephew, Andover, Mass, USA) or Ethibond no. 2 sutures (Ethicon Inc., Somerville, NJ, USA) tied over an AO bicortical screw (AO Foundation, Davos, Switzerland) with a washer on the tibial side. The existence of concomitant injuries to the menisci or the articular cartilage was determined intraoperatively. In the event of meniscal injuries regarded as suitable for suturing, an all-inside arthroscopic technique using a FAST-FIX suture anchor device (Smith and Nephew, Andover, Mass, USA) was used for tears located in the dorsal or central part of the meniscus. Meniscal lesions in the anterior part were repaired with an outside-in technique using PDS 0 (Ethicon, Inc, Sommerville, NJ, USA).

### Rehabilitation

All the patients followed a standardized rehabilitation protocol. Immediate full weight-bearing and full range of motion (ROM) were encouraged if tolerated, for patients with meniscal resection or isolated ACL reconstruction. A reduction in postoperative swelling, gait correction, and the recovery of ROM was the aims in the early phase of rehabilitation. In the event of meniscal repair, patients were recommended a hinged knee brace with flexion limited from 0° to 30° for the first 2 weeks, from 0° to 60° for the 3rd and 4th weeks, and from 0° to 90° for the 5th and 6th weeks. Quadriceps strengthening was restricted to closed kinetic chain exercises in the first 3 months postoperatively. The timing of return to sports was individualized, depending on the type of activity and knee function. However, return to sports earlier than 6 months postoperatively was discouraged.

### Physical examination and follow-up

Physical examinations were performed preoperatively and 6 months postoperatively. Range of motion was assessed using a goniometer. To analyze the influence of a gradual increase in contralateral knee hyperextension in the ATT of the ACL-injured knee, four subgroups were created. The subgroups constituted patients with no hyperextension (Group A ≤ 0°), mild (Group B 1°–5°), moderate (Group C 6°–10°), and severe hyperextension (Group D > 10°). A 5° increment interval was chosen to detect a potential trend. The degree of extension of the contralateral knee was used to determine subgroup placement, since the contralateral knee was regarded as more representative of the preinjury knee extension level of the ACL-injured knee. Anterior tibial translation was measured preoperatively and at the 6-month postoperative follow-up using the KT-1000 arthrometer (MEDmetric Corp, San Diego, CA, USA). A 134-N anterior load was applied with the knee at 20 degrees of flexion. At least three measurements were made on each knee and the median value was registered. The anterior tibial displacement was expressed in millimeters. Differences in ATT between the groups were also determined by observing side-to-side difference, meaning the difference in ATT between the injured and the contralateral knee. Finally, by analyzing the changes in pre- to postoperative side-to-side differences in ATT, a comparison between the subgroups regarding postoperative ATT reduction was possible.

### Data management

All surgeons performing ACL reconstructions at the Capio Artro Clinic are obliged to report surgical and medical history data to the local register to proceed to the patient’s specific medical record. This organization ensures complete coverage of data relating to the previous contra- or ipsilateral surgery, concomitant knee injuries, graft choice, fixation method, and other potential simultaneous interventions. The study was approved by the Regional Ethics Committee at Karolinska Institutet (2016/1613-31/2).

### Statistical analysis

Categorical variables were presented with numbers and percentages. Continuous variables were presented using means, standard deviations, medians, and range. Demographic variables were analyzed using Fischer’s exact test and the Mann–Whitney *U* test for dichotomous and continuous variables, respectively. The distribution of concomitant meniscal and cartilage injuries was analyzed using multivariate logistic regression analysis, adjusted for age and gender. Similarly, in the analysis of KT-1000 parameters, multivariate logistic regression was used to detect differences between the reference group (Group A) and the other subgroups. Multivariate analysis of the KT-1000 analysis was chosen to enable the adjustment of the following covariates: age, gender, graft choice, and meniscal injuries. Statistical significance was set at 0.05.

## Results

**Fig. 1:**
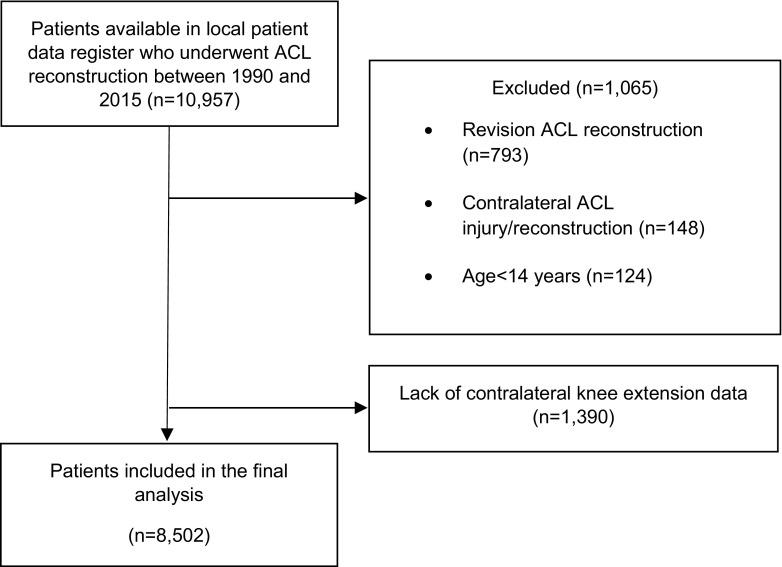
Flowchart of included patients. *ACL* anterior cruciate ligament, *n* number of patients

A total of 10,957 patients were available in the local patient data register, of which 1065 (9.7%) patients were excluded. The most frequent reason for exclusion was revision ACL surgery (793 patients, 74.5%), followed by contralateral ACL injury (148 patients, 13.9%) and age below 14 years at the time of surgery (124 patients, 11.6%, Fig. 1).

A total of 8502 (77.6%) patients had ROM data for the contralateral healthy knee available and were included in the final analysis. Demographic parameters for the analyzed subgroups, based on the degree of contralateral knee hyperextension, are presented in Table 1. The largest subgroup was composed of patients with no hyperextension (Group A 4335 patients). Groups B, C, and D contained 3331, 771, and 65 patients, respectively. Patients with the highest degree of hyperextension (Group D) had the youngest mean age (25.6 ± 8.1 years), whereas patients with no hyperextension (Group A) had the oldest mean age (29 ± 10.2 years, *p* = 0.013). There was a higher rate of females in the subgroups with hyperextension (Group B 46.6%, *p* < 0.0001, Group C 47.9%, *p* = 0.0003, Group D 47.7%, *p* = 0.34) compared with the subgroup with no hyperextension (Group A 39.7%). A patellar tendon autograft was more commonly used in patients with hyperextension (Group B 38.4%, *p* < 0.0001, Group C 42.5%, *p* < 0.0001, Group D 50.8%, *p* < 0.0001) than in patients without (Group A 26.4%, Table 1). An analysis of meniscal injuries, including injuries to both the medial and the lateral menisci, showed that injuries were proportionally more frequent in patients with no hyperextension (Group A 34.1%) compared with patients with an increasing degree of hyperextension (Group B 27.1%, *p* < 0.0001, Group C 27.0%, *p* = 0.0003, Group D 18.5%, *p* = 0.012) (Table 2).

**Table 1:** Demographics of contralateral knee extension subgroups

Outcome variables	Subgroups based on contralateral knee extension									
	Group A (HE ≤ 0°)	Group B (HE 1°–5°)			Group C (HE 6°–10°)			Group D (HE > 10°)		
			Diff between groups, mean (95% CI)	*p* value		Diff between groups, mean (95% CI)	*p* value		Diff between groups, mean (95% CI)	*p* value
Age, years	29 (10.2) 28 (14; 90) *n* = 4335	27.5 (9.6) 26 (14; 66) *n* = 3331	1.48 (1.03; 1.91)	< 0.0001	26.8 (9.4) 26 (14; 79) *n* = 771	2.19 (1.46; 2.91)	< 0.0001	25.6 (8.1) 24 (15; 48) *n* = 65	3.37 (1.34; 5.34)	0.013
Patient sex										
Male	2612 (60.3%)	1778 (53.4%)	6.9 (4.6; 9.1)		402 (52.1%)	8.1 (4.2; 12.0)		34 (52.3%)	7.9 (− 5.1; 21.0)	
Female	1723 (39.7%)	1553 (46.6%)	− 6.9 (− 9.1; − 4.6)	< 0.0001	369 (47.9%)	− 8.1 (− 12.0; − 4.2)	0.0003	31 (47.7%)	− 7.9 (− 21.0; 5.1)	ns
Tendon										
Patellar tendon	1145 (26.4%)	1278 (38.4%)	− 12.0 (− 14.1; − 9.8)		328 (42.5%)	− 16.1 (− 19.9; − 12.3)		33 (50.8%)	− 24.4 (− 37.4; − 11.4)	
Hamstring tendon	3190 (73.6%)	2053 (61.6%)	12.0 (9.8; 14.1)	< 0.0001	443 (57.5%)	16.1 (12.3; 19.9)	< 0.0001	32 (49.2%)	24.4 (11.4; 37.4)	< 0.0001

For categorical variables, *n* (%) is presented

For continuous variables, the mean (SD)/median (min; max)/*n* = is presented

Comparisons were made between control Group A and the other groups using Fisher’s exact test (lowest one-sided *p* value multiplied by 2) for dichotomous variables and the Mann–Whitney *U* test for continuous variables

*Diff* difference, *HE* hyperextension

**Table 2:** Concomitant meniscal and chondral injuries

Outcome variables	Subgroups based on contralateral knee extension									
	Group A (HE ≤ 0°)	Group B (HE 1°–5°)			Group C (HE 6°–10°)			Group D (HE > 10°)		
			Diff between groups, mean (95% CI)	*p* value		Diff between groups, mean (95% CI)	*p* value		Diff between groups, mean (95% CI)	*p* value
Cartilage injury										
Yes	678 (15.6%)	404 (12.1%)	3.5 (1.9; 5.1)		92 (11.9%)	3.7 (1.1; 6.3)		7 (10.8%)	4.9 (− 3.5; 13.3)	
No	3657 (84.4%)	2927 (87.9%)	− 3.5 (− 5.1; − 1.9)	0.0015	679 (88.1%)	− 3.7 (− 6.3; − 1.1)	ns	58 (89.2%)	− 4.9 (− 13.3; 3.5)	ns
Medial meniscus injury										
Yes	911 (21.0%)	527 (15.8%)	5.2 (3.4; 7.0)		124 (16.1%)	4.9 (2.0; 7.9)		7 (10.8%)	10.2 (1.8; 18.7)	
No	3424 (79.0%)	2804 (84.2%)	− 5.2 (− 7.0; − 3.4)	< 0.0001	647 (83.9%)	− 4.9 (− 7.9; − 2.0)	0.0062	58 (89.2%)	− 10.2 (− 18.7; − 1.8)	ns
Lateral meniscus injury										
Yes	827 (19.1%)	527 (15.8%)	3.3 (1.5; 5.0)		115 (14.9%)	4.2 (1.3; 7.0)		7 (10.8%)	8.3 (− 0.1; 16.7)	
No	3508 (80.9%)	2804 (84.2%)	− 3.3 (− 5.0; − 1.5)	0.0002	656 (85.1%)	− 4.2 (− 7.0; − 1.3)	0.0043	58 (89.2%)	− 8.3 (− 16.7; 0.1)	ns
Meniscal injury, dichotomous										
Yes	1479 (34.1%)	903 (27.1%)	7.0 (4.9; 9.1)		208 (27.0%)	7.1 (3.6; 10.7)		12 (18.5%)	15.7 (5.3; 26.0)	
No	2856 (65.9%)	2428 (72.9%)	− 7.0 (− 9.1; − 4.9)	< 0.0001	563 (73.0%)	− 7.1 (− 10.7; − 3.6)	0.0003	53 (81.5%)	− 15.7 (− 26.0; − 5.3)	0.012

For categorical variables, *n* (%) is presented

For continuous variables, the mean (SD)/median (min; max)/*n* = is presented

Comparisons were made between control Group A and the other groups using multivariate logistic regression analysis adjusting for age and gender

*Diff* difference, *HE* hyperextension

The mean preoperative ATT of the healthy contralateral knee was significantly greater for Groups B and C compared with Group A (*p* < 0.0001 and *p* = 0.0002, respectively). Group D did not differ in terms of ATT when compared with Group A (ns). Similar results were seen for the ACL-injured knee preoperatively, with a significantly greater ATT for Groups B and C compared with Group A (Table 2). Multivariate analysis of the ATT of the injured knee 6 months postoperatively revealed a gradual increase in ATT, from 8.2 mm (Group A) to 8.5 (Group B, *p* < 0.0001), 8.5 (Group C, *p* < 0.035), and 9.1 (Group D, ns). An analysis of preoperative or 6-month postoperative differences in side-to-side measurements of the injured knee did not reveal any significant differences between the subgroups (Table 3).

**Table 3:** KT-1000 outcome variables with regard to contralateral knee extension subgroups

Outcome variables	Subgroups based on contralateral knee extension									
	Group A (HE ≤ 0°)	Group B (HE 1°–5°)			Group C (HE 6°–10°)			Group D (HE > 10°)		
	Mean (mm)	Mean (mm)	95% CI	*p* value	Mean (mm)	95% CI	*p* value	Mean (mm)	95% CI	*p* value
KT-1000 preoperative, contralateral knee	6.5	6.8	(0.14–0.42)	< 0.0001	6.9	(0.17–0.68)	0.0002	6.3	(− 1.01–0.60)	ns
KT-1000 preoperative, injured knee	10.1	10.3	(0.06–0.42)	0.0055	10.6	(0.23–0.89)	0.0004	10.0	(− 1.08–0.96)	ns
KT-1000 6-month postoperative, injured knee	8.2	8.5	(0.13–0.45)	< 0.0001	8.5	(0.02–0.60)	0.035	9.1	(− 0.08–1.77)	ns
KT-1000 preoperative, side-to-side difference	3.5	3.5	(− 0.22–0.14)	ns	3.7	(− 0.19–0.45)	ns	3.7	(− 0.88–1.14)	ns
KT-1000 6-month postoperative, side-to-side difference	1.7	1.8	(− 0.06–0.23)	ns	1.7	(− 0.21–0.30)	ns	2.4	(− 0.08–1.56)	ns
KT-1000 reduction from preoperative to postoperative side-to-side difference	− 1.8	− 1.7	(− 0.09–0.28)	ns	− 1.9	(− 0.41–0.24)	ns	− 1.4	(− 0.62–1.43)	ns

Analysis was adjusted for age, gender, type of graft, and meniscal injury (dichotomous). Comparisons were made between control Group A and the other groups using multivariate logistic regression analysis

*CI* confidence interval, *HE* hyperextension

## Discussion

The main finding in this study was the significantly higher level of ATT measured in the ACL-injured knee in patients with contralateral knee hyperextension, thereby confirming the hypotheses stated in the introduction. Patients with mild and moderate contralateral knee hyperextension showed a significant increase in ATT in the ACL-injured knee compared with patients with no hyperextension, both pre- and postoperatively. Conversely, the group with severe hyperextension, corresponding to a level of hyperextension equivalent to a Beighton score point, did not reveal significantly higher preoperative ATT in either the injured or the contralateral knee. A tendency towards an increase in ATT was observed at the 6-month follow-up in patients with severe hyperextension, presenting with the highest mean ATT of all groups. Analyzing the same group, a similar tendency was observed for the side-to-side difference analysis, indicating an increase in postoperative ATT compared with the reference group. The relatively small number of patients in the group with severe hyperextension is a possible explanation for the analyses not reaching statistical significance, making the analysis underpowered. The reduction in pre- to postoperative ATT was the same after ACL reconstruction in all subgroups. An acceptable ATT can thus also be reached in patients with hyperextension after ACL reconstruction, at least in the short term.

Although conclusions cannot be drawn from this study alone, the trend towards an increase in the postoperative ATT in the ACL-injured knee raises a suspicion of inferior quality in the autograft or inferior graft remodeling in patients with hypermobility. An important study by Larson et al. [15] demonstrated that patients with joint hypermobility run an elevated risk of both graft re-rupture and contralateral ACL rupture. Moreover, joint hypermobility is thought to be caused by alterations in the connective tissue [20]. It is, therefore, reasonable to assume that the higher degree of ATT observed in patients with contralateral knee hyperextension may be attributable in part to suboptimal conditions in the connective tissues of both the graft and the surrounding secondary stabilizers, such as the joint capsule and the menisci.

It was hypothesized that hyperextension would be associated with an increased risk of associated injuries, but, in actual fact, meniscal injuries were clearly less common with increasing contralateral knee hyperextension. Knowing that joint hypermobility is a risk factor for ACL injury [18, 19, 21, 27], it may seem logical to assume that an increasing incidence of concomitant injures would also be observed in patients with knee hyperextension. However, one possible explanation could be that the knees of patients with no joint hypermobility are more resilient and more severe traumas are required, with increasing torque acting at the knee joint at the time of injury, to rupture their ACL. More severe traumas would also increase the risk of concomitant intra-articular injuries. Individuals with less joint hypermobility would, therefore, hypothetically, not sustain an ACL injury when exposed to the same amount of force as a hypermobile individual.

It the present study, it was obvious that the use of PT autografts was increasingly more common in patients with higher degrees of contralateral knee hyperextension and this factor was, therefore, adjusted for in the statistical analysis. Previously, Kim et al. [11, 12, 14] have demonstrated that a PT autograft is superior to an HT autograft in terms of knee stability and function in patients with knee hyperextension. On the same subject, Benner et al. [5] studied patients with knee hyperextension of the ACL-injured knee and were not able to observe any differences in ACL graft rupture or subjective outcome depending on the level of knee hyperextension, therefore, assuming that the use of a PT autograft was a good alternative. The reason for the dominance of the PT autograft in hyperextending knees observed in the present study was not intentional at an organized level, meaning that there were no institutional recommendations for using PT autografts in this category of patients. However, individual surgeons may have preferred the PT autograft, considering the increased preoperative ATT seen in these patients, with the knowledge that the PT autograft is thought to be better at reducing ATT [17].

As mentioned, patients with knee hyperextension and GJH are susceptible to ACL injury [18, 19, 21, 27], making them a priority for further research. The present study reveals new data enhancing our understanding of anterior knee laxity in patients with knee hyperextension. Future studies are needed to investigate potential differences in ATT in this patient group over longer follow-up periods. The question of the potential influence of knee hyperextension on rotatory knee laxity also remains to be answered in future studies. Moreover, the newly established inverse association between contralateral knee hyperextension and injuries to the menisci and articular cartilage needs to be further scrutinized.

One limitation of the present study is the short follow-up period of 6 months. After 6 months, many patients will not have returned to their preoperative level of activity and the strength of the graft will not have been tested to the limit of its ability and tensile strength. As a result, this study possibly underestimates postoperative long-term ATT and the results should not be regarded as representative for patients who have returned to sports. The difference in the risk of graft rupture or long-term graft failure could not be assessed. Moreover, the differences in ATT observed between the groups are small in absolute numbers and the differences between the means of the groups are within the margin of error for the KT-1000 arthrometer in examinations of individual patients [24]. At the same time, since analyses are made at group level, containing a large number of patients, the results indicate a progressive increase in ATT with increasing degrees of contralateral knee hyperextension.

In summary, this study underlines the greater risk of having increased ATT for patients with hypermobile joints. Since it has been shown that the use of PT autograft, compared with HT autograft, is associated with less postoperative ATT [17, 23], the use of this graft-type could be recommended for patients with significant contralateral knee hyperextension to lower the risk of having a greater postoperative anteroposterior laxity.

## Conclusion

Contralateral knee hyperextension is associated with greater pre- and postoperative ATT in the ACL-injured knee. In patients with contralateral knee hyperextension, concomitant injuries to the menisci are less frequent. Surgeons should consider grafts with superior properties regarding postoperative anteroposterior laxity to patients with contralateral knee hyperextension.
